# Remediation of Hg-Contaminated Groundwater via Adsorption on Supramolecular Polymers in Batch Process and Column Test

**DOI:** 10.3390/molecules30071406

**Published:** 2025-03-21

**Authors:** Zongwu Wang, Wei Liu, Xiaoyan Sun, Qing Zhang, Jiapu Ji, Yimeng Yan, Jianhui Sun

**Affiliations:** 1 Department of Environment Engineering, Yellow River Conservancy Technical Institute, Kaifeng Engineering Research Center for Municipal Wastewater Treatment, Kaifeng 475004, China; 2 Product Quality Inspection and Testing Center of Kaifeng, Kaifeng 475004, China; 3 MOE Key Laboratory of Yellow River and Huai River Water Environmental and Pollution Control, School of Environment, Henan Normal University, Xinxiang 453007, China

**Keywords:** supramolecular polymers, groundwater, mercury, remediation

## Abstract

Mercury contamination in groundwater seriously affects human health and ecosystem security. The remediation of Hg-contaminated groundwater remains a challenging task. The applicability of an as-synthesized supramolecular polymer (SP) for low-concentration mercury in a high-salinity groundwater matrix has been verified through a batch process and column test. The remediation of mercury-contaminated groundwater, particularly in complex high-salinity environments, represents a significant and enduring challenge in environmental science. The batch test study demonstrated that the SP can efficiently adsorb Hg from groundwater with superior selectivity and a high uptake capacity (up to 926.1 ± 165.3 mg g^−1^). Increasing the pH and dissolved organic matter (DOM) and reducing the ionic strength can facilitate Hg adsorption; the coexistence of heavy metal ions slightly weakens the removal. In terms of its performance as a permeable reactive barrier, the SP can intercept Hg in flowing groundwater with a capacity of up to 3187 mg g^−1^. A low influent mercury concentration, low pore velocity, and high SP dosage can effectively extend the breakthrough time in column tests. Additionally, the Yan model (*R*^2^ = 0.960−0.989) can accurately depict the whole dynamic interception process (150 PVs) of SPs in a fixed column, and the Adams–Bohart model (*R*^2^ = 0.916−0.964) describes the initial stage (≤35 PVs) well. Considering the functional group in the SP and the Hg species in groundwater, complexation, electrostatic attraction, ion exchange, and precipitation/co-precipitation are the plausible mechanisms for mercury removal based on the characterization results of scanning electron microscopy (SEM), X-ray photoelectron spectroscopy (XPS), and Fourier transform infrared spectrometer (FT-IR). These impressive features render the SP a promising candidate for the remediation of trace Hg in saline groundwater using permeable reactive barrier (PRB) technology.

## 1. Introduction

Mercury (Hg) is one of the most toxic persistent heavy metals, and its presence poses a serious threat to the ecological environment and human health. The reduction of mercury in the environment and health has become a global management and technology focus [1,2]. Among the various valence states of mercury, Hg^2+^ is the most abundant. Incidents of mercury contamination in groundwater have been reported worldwide, with Hg concentrations in groundwater ranging from 66 μg L^−1^ to 2005 μg L^−1^ [3,4,5,6]. Furthermore, the established maximum contaminant level (MCL) established toward Hg in drinking water is 2 μg L^−1^ (USEPA, 2010), and the World Health Organization (WHO) guideline for Hg in drinking water is 1 μg L^−1^. Groundwater is an important source of drinking water and irrigation water for agriculture. The guideline value of Hg with respect to groundwater for drinking in China is 1 μg L^−1^ [7]. Therefore, it is ecologically and strategically imperative to explore cost-effective technologies to address this global dilemma.

Groundwater is rich in minerals, and the major species among them (${\mathrm{Na}}^{+}$, ${\mathrm{Ca}}^{2+}$, ${\mathrm{Mg}}^{2+}$, $K^{+}$, ${\mathrm{HCO}}_{3}^{-}$, ${\mathrm{SO}}_{4}^{2-}$, ${\mathrm{Cl}}^{-}$, and ${\mathrm{NO}}_{3}^{-}$) always account for >99% of the solute content [8], leading to high ionic strength and high salinity. The presence of multiple ions affects the form of mercury [9], which is related to the fluidity and toxicity of mercury. In addition, the water quality and hydrological conditions of groundwater, such as the pH, mercury concentration, dissolved organic matter (DOM), and pore velocity, also have a significant influence on the presence and migration of mercury [10,11,12]. The difficulty of removing mercury from groundwater is compounded by the complex substrate [2,9]. Among the various methods of removing contaminants from flowing groundwater, permeable reactive barriers (PRBs) stand out for their simple operation, effective in situ interception and ease of scale-up from laboratory to industrial scale [13,14]. Reactive sorbents with high efficiency and low cost, filled in PRBs to trap the migrating contaminants in groundwater, have become the bottleneck in promoting the applications of PRBs. To date, persistent efforts have been made to fabricate reactive engineered materials for the in situ remediation of heavy metal contaminated groundwater, such as activated carbon, resins, zeolites, clay, zero-valent iron (ZVI), graphene oxide (GO), solid waste residue, chitosan, chelating ligands, metal organic frameworks (MOFs), titanate nanotubes, etc. [13,15,16,17,18,19,20,21,22,23,24,25]. Limited by the low sorption capacity of activated carbon, resins, zeolites, chitosan, and clay for mercury; the chemical instability of ZVI in groundwater; the potential decrease in permeability of GO and solid waste residue at a given flow rate; and the environmentally unfriendly and high cost of chelating ligands and MOFs, a reliable sorbent with high selectivity and capacity is highly desirable for the remediation of Hg-contaminated groundwater in PRBs.

As an environment-friendly material, polymeric material has become one of the most promising candidates, benefiting from the excellent controllable chemical functionality, dimensional stability, and extraordinary adsorption property [26,27]. The development of an innovative synthesis method and the screening of suitable raw materials for the production of highly efficient polymeric materials is, therefore, the crucial link. Trithiocyanuric acid (TTCA), the main precursor of the commercial adsorbent TMT15, which is limited in practice by aggregation, pH limitation, and difficult recovery, has a strong complexing ability of sulfur ligands with many soft Lewis acid metal ions (Pb^2+^, Hg^2+^, etc.) [27,28,29,30,31,32]. The organodisulfide polymer derived from TTCA has the potential to be an excellent heavy metal scavenger but suffers from the use of toxic reagents, complex synthesis steps, and limited yield, which prevent large-scale application [33,34,35,36]. Recent work has shown that melamine (MA)-based dendrimers also exhibit high scavenging capacity due to the complexation between –NH_2_ and heavy metals [37,38]. It is still essential to find a way to combine the advantages of TTCA and MA while avoiding their disadvantages. Few attempts have been made to enrich precious metals from aqueous substances by the sequential addition of TTCA and MA [39,40]. Furthermore, a chemically stable supramolecular polymer (SP), through non-covalent combination, has been synthesized through the high hydrogen bonding of N–H···S and N–H···N via the reorganization of MA and TTCA at the molecular level [41,42,43,44,45,46]. A magnetic supramolecular polymer fabricated via hydrogen bonding showed excellent capture ability for Pb and Cr in wastewater based on our previous study [47,48]. However, to the best of our knowledge, the investigation of the SP as a reactive material in PRBs for Hg^2+^ removal in groundwater has not yet been reported.

Motivated by the above research gaps, we systematically investigated the batch and column performance of the SP to assess its applicability in removing Hg^2+^ from simulated groundwater. The objectives of this study were (1) to explore the application of the SP to mitigate mercury concentrations through batch experiments by investigating the effects of the pH, dosage, dissolved organic matter (DOM), ionic strength, and coexisting ions on the adsorption performance of Hg^2+^ in groundwater; (2) to describe the breakthrough curves of the SP toward Hg^2+^ using typical models (Adams–Bohart, Thomas, Yoon–Nelson, and Yan) and to investigate the effects of operation conditions (including the influent Hg^2+^ concentration, pore velocity, and sorbent dosage) for the SP in PRBs via a fixed-bed column test; and (3) to reveal the plausible mechanism of Hg^2+^ adsorption on the SP based on the results of routine and column experiments coupled with component characterization.

## 2. Results and Discussion

### 2.1. Material Characterization

**Figure 1:**
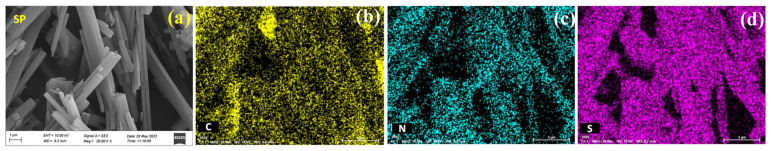
SEM images of SP (**a**); SEM mapping of elements C (**b**), N (**c**), and S (**d**) for SP.

**Figure 2:**
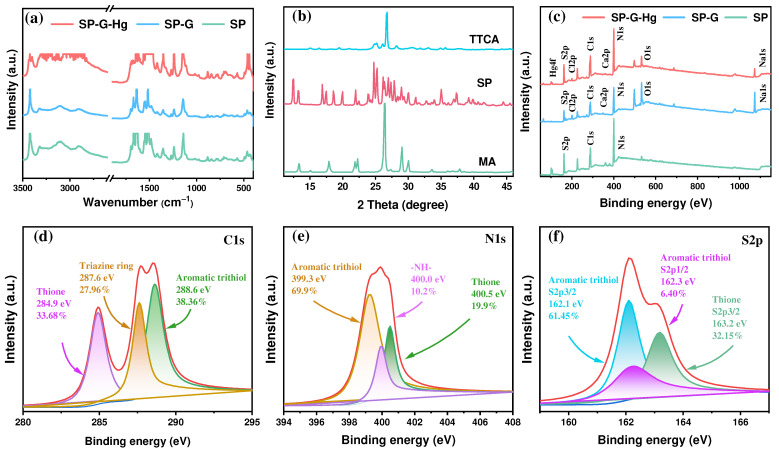
FT−IR (**a**), XRD (**b**), and XPS (**c**) patterns of the as-prepared samples. SP-G (SP after adsorption in the simulated groundwater without Hg) and SP-G-Hg (SP after adsorption in the simulated groundwater with Hg); high-resolution XPS spectra of (**d**) C1s, (**e**) N1s, and (**f**) S2p for the SP.

The SP obviously exhibited a distinct rod-like structure and smooth surface (Figure 1 and Figure S1), and the uniformly distributed presence of C, N, and S atoms on the surface was observed. In the FT-IR spectra (Figure 2a), the N–H stretching vibration that correspond to hydrogen bonding (N–H···S and N–H···N) are centered at 3420 cm^−1^. The signal at 880 cm^−1^ is attributed to the bending vibration of heterocycles in the polymer structure of the SP. The peaks around 1633 cm^−1^, 1142 cm^−1^, and 780 cm^−1^ correspond to the thione, the C=S stretching vibration, and the thiazine ring vibration, respectively [28,47]. The peak at 3420 cm⁻^1^ in the SP provides clear evidence of hydrogen bonding interactions.

The SP also has high crystallinity (Figure 2b), confirming the formation of a supramolecular polymer. Compared with the XRD patterns of MA and TTCA, there are three signals at 18.57°, 13.19°, and 12.25°, which can be attributed to in-planar packing, and the peak at 24.76° is indexed to the graphite-like structure of SP sheets [42,49,50]. The crystallinity indices (CrI) from XRD for TTCA, SP, and MA are 0.773, 0.579, and 0.797, respectively. As shown in Figure 2c, three signals centered at 288.1, 399.6, and 162.0 eV represent the coexistence of C, N, and S elements in the SP, corresponding to C1s, N1s, and S2p, respectively [51,52]. In the high−resolution XPS spectra of C1s (Figure 2d), there are three forms, including the thione (33.68%, 284.9 eV), the thiazine ring (29.76%, 287.6 eV), and the aromatic trithiol (38.36%, 288.6 eV) in the SP. For the N1s XPS spectra (Figure 2e), signals at 399.3 eV (69.99%), 400.0 eV (10.20%), and 400.5 eV (10.20%) represent the aromatic trithiol, the –NH–, and the thione, respectively [28,51,53,54]. The peaks situated at 162.1 and 162.3 eV fit the spectra of S2p3/2 (61.45%) and S2p1/2 (6.40%) in the aromatic trithiol and that of 163.2 eV indicates the S2p3/2 in the thione (Figure 2f) [34,55,56].

### 2.2. Interfacial Processes of Hg in Groundwater

**Figure 3:**
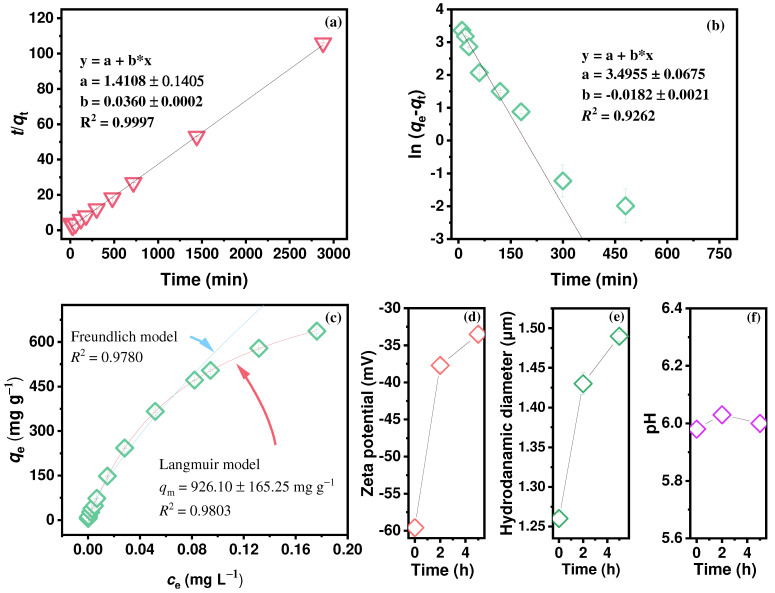
Fitting of the pseudo-second-order kinetic model (**a**), the pseudo-first-order model (**b**), and isotherms (**c**) in the simulated groundwater for mercury adsorption onto the SP (*m* = 2.0 mg L^−1^; *C*_0_ = 0.01–1.50 mg L^−1^; T = 298 K); changes in the (**d**) zeta potential, (**e**) hydrodynamic diameter, and (**f**) pH (*m* = 2.0 mg L^−1^; *C*_0_ = 0.08 mg L^−1^; T = 298 K).

To understand the interfacial processes of Hg adsorption in groundwater, the pseudo-first- and pseudo-second-order kinetic models were used [52,57,58,59]. The sorption kinetics was analyzed using both the pseudo-first- and pseudo-second-order kinetic models to provide a comprehensive understanding of the interfacial processes (Text S1). The SP exhibited a higher Hg removal efficiency (96.39%), superior adsorption capacity (38.63 ± 0.03 mg g^−1^), and shorter equilibration time (5 h) (Figure S2a). The uniform dispersion of N and S in the SP significantly facilitated the adsorption to Hg (Figure 1c,d). In addition, the fitting was conducted through the plots of ln (*q*_e_ − *q*_t_) vs. *t*, *t*/*q*_t_ vs. *t* (Figure 3a,b). The *R*^2^ (0.9996) of pseudo-second-order equation for Hg^2+^ removal was better than that of pseudo-first model (0.9262). In addition, the equilibrium adsorption capacity *q*_e_ (39.22 ± 0.31 mg g^−1^) based on the fitted results of the pseudo-second-order kinetics is approximately equal to the experimental result (Table S1). The pseudo-second-order model is based on the assumption that the rate-limiting step may be chemical adsorption or chemisorption, involving electron sharing or electron transfer between the sorbate and the sorbent. The better fit of the pseudo-second-order kinetic model suggests that the rate-limiting step in the adsorption process may be chemisorption through valency forces involving electron sharing between mercury and the SP on heterogeneous surfaces [52,60].

To further understand the effect of the concentration on adsorption, varying initial concentrations *C*_0_ (0.01–1.50 mg L^−1^) were studied. An increase in the adsorption capacity *q*_e_ was accompanied by an increase in the *C*_0_, which can be attributed to the stronger ability of overcoming mass-transfer resistance resulting from the concentration gradient. The *q*_t_ increases rapidly with an increasing time before 300 min, and it starts to stabilize after 300 min (Figure S2a). By contrast, the removal efficiency by the SP decreased significantly (Figure S2b). Langmuir isotherm and Freundlich isotherm models (Text S2) were used to depict the adsorption equilibrium [61,62]. The nonlinear fitting of *q*_e_ vs. *c*_e_ is shown in Figure 3c. The Langmuir isotherm (*R*^2^ = 0.9803) matched the adsorption data better than the Freundlich isotherm (*R*^2^ = 0.9780) (Table S2). The Freundlich model predicts nonideal sorption on heterogeneous or multilayer surfaces, but the Langmuir model can be applied to a constant monolayer-sorption capacity. This indicates that Hg was mainly adsorbed via monolayer adsorption on heterogeneous surfaces of the SP during the interfacial process [52,62]. Furthermore, the *q*_m_ of the SP is up to 926.1 ± 165.3 mg g^−1^ due to the rich –SH and –NH_2_ in the supramolecular polymer (SP), which is higher than that of the most recently reported capacities (Table 1).

To better understand the temperature effect on mercury adsorption, thermodynamic studies were performed. It can be seen that a higher temperature is favorable for Hg removal (Figure S2b), indicating that the adsorption process is endothermic. Furthermore, the negative free energy changes (ΔG) and the decreasing trend with an increasing temperature indicate the spontaneity of the adsorption (Table S3). The positive enthalpy change (ΔH) shows that the adsorption process is endothermic, and the positive entropy change (ΔS) suggests the increasing randomness at the interface between Hg and the SP during the adsorption. This also indicates that the adsorption is likely to be chemisorption, which is consistent with the kinetic analysis.

For the selected simulated groundwater, Hg(OH)_2_, HgClOH (aq), HgCl_2_ (aq), and ${\mathrm{HgOHCO}}_{3}^{-}$ (aq) were the predominant Hg species, with proportions of 45.9%, 40.4%, 7.1%, and 5.7%, respectively [2]. ${\mathrm{Ca}}^{2+}$, ${\mathrm{CO}}_{3}^{2-}$, and ${\mathrm{SO}}_{4}^{2-}$ were the major anions and cations, which may lead to the presence of CaCO_3_ precipitation. Precipitation/co-precipitation of Hg^2+^ and ${\mathrm{Ca}}^{2+}$ could occur. The surface charge of SP is negative (Figure 3d), and the positive (Hg^2+^, HgCl^+^, and HgOH^+^) or uncharged charge (Hg(OH)_2_, HgClOH, and HgCl_2_) could be adsorbed on the SP via electrostatic attraction. Therefore, the SP could selectively adsorb mercury species before calcium species, resulting in an increase in both the zeta potential (from −59.6 mV to −33.5 mV) and hydraulic diameter (from 1.26 μm to 1.49 μm) (Figure 3d,e). The pH change throughout the process is almost negligible (Figure 3f).

### 2.3. Effects of pH, Ionic Strength, DOM, and Coexisting Ions

**Figure 4:**
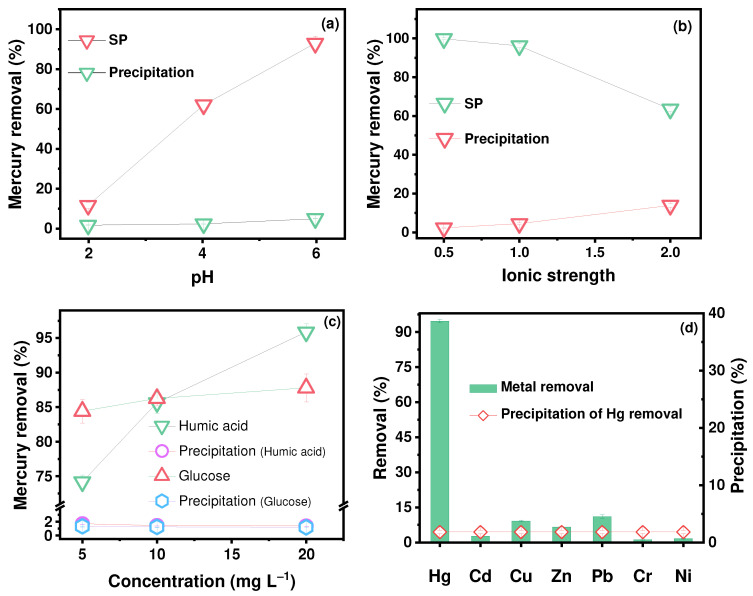
Effects of the (**a**) pH, (**b**) ionic strength, (**c**) DOM, and (**d**) coexisting heavy metal ions for Hg removal performance from a mixed solution of heavy metal ions. Experimental conditions for (**d**): 2.0 mg L^−1^ for the SP, *C*_0_ = 4.0 × 10^−4^ mmol L^−1^ for Hg and each coexisting heavy metal, and a mass concentration of 0.08 mg L^−1^ for Hg^2+^.

**Figure 5:**
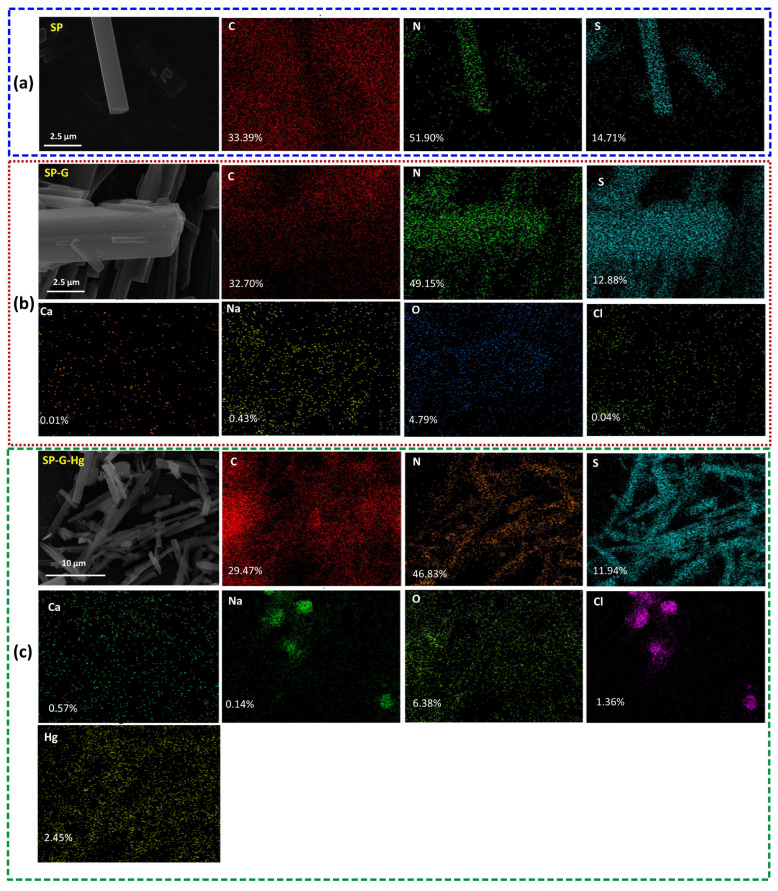
SEM-mapping images of individual elements C, N, and S in the SP (**a**), C, N, S, Ca, Na, O, and Cl in the SP-G (**b**), and C, N, S, Ca, Na, O, Cl, and Hg in the SP-G-Hg (**c**). The numbers in the mapping represent atomic percentages.

The efficiency of the mercury removal by the SP increased from 11.66% to 92.94% in a pH range from 2.0 to 6.0 (Figure 4a). The pH can both vary the surface charge of the SP and affect the mercury species in simulated groundwater [65,66]. At pH < 4.0, H^+^ occupies partial active sites on the SP. This can lead to a weakening of the negative charges, weakening the electrostatic attraction of the SP to mercury and causing competition (e.g., cation–π interaction and ligand exchange) between H^+^ and Hg^2+^. With an increasing pH, the decrease in H^+^, the increase in negative charge, and the enhancement of mercury precipitation/co-precipitation result in a significant increase in the Hg removal rate and a small increase in precipitate removal (from 1.75% to 5.03%). Higher ionic strength hindered the Hg^2+^ removal (Figure 4b). Reasons for this include competition from other cations (Ca^2+^ and Na^+^) for adsorption sites on the SP, shielding caused by a high salt content with a neutral electrical charge (e.g., NaCl, Na_2_SO_4_, CaCl_2_, and NaHCO_3_) between Hg and the SP, the strong inhibition of precipitation on interfaces, and the limitations imposed by C1^−^ due to metal chlorides [2,65,67,68,69,70]. Regarding the role of the groundwater matrix (e.g., Ca^2+^, Na^+^), it can be seen that a certain amount of Ca (0.01%) and Na (0.43%) is adsorbed onto the SP in the groundwater without mercury (Figure 5a,b). However, in the Hg-contaminated groundwater, perhaps due to competition or subsequent ion exchange, the Na content decrease to 0.14% (2.45% for Hg), indicating that the presence of Na^+^ has a negative but negligible effect (Figure 5c). However, as the Hg content increases to 2.5%, Ca also shows an increase (from 0.01% to 0.57%), which may be due to precipitation or co-precipitation, indicating that the presence of Ca^2+^ can promote mercury removal. This shows that the SP has good applicability to real-world conditions.

As a typical macromolecular DOM, humic acid (HA) has a strong affinity for Hg due to the functional groups in HA (e.g., –OH, –NH_2_, –COOH, and –SH) [67,71]. Therefore, mercury in groundwater was scavenged by both the SP and HA through complexation. At low HA concentrations, HA in solution will very readily complex Hg in groundwater. This leads directly to a decrease in the removal of Hg by the SP. With an increasing HA concentration, some HA complexed with mercury in groundwater was adsorbed on the SP, which resulted in a significant enhancement of Hg removal from the aqueous solution to the SP (from 74.19% to 95.87%) (Figure 4c). Increasing the concentration of glucose (GLu) had little effect on interfacial adsorption. For both HA and GLu, Hg precipitation (1.19%−1.69%) showed robust resistance to changes in the concentration. In the presence of coexisting heavy metal ions (Cd, Cu, Zn, Pb, Cr, and Ni), the selectivity of the SP for mercury removal in simulated groundwater was investigated (Figure 4d). It can be seen that SP removal slightly decreased but remained relatively high (94.63%) compared to the six other metal species (1.17−11.11%). This suggests that despite the competition for active sites between seven heavy metals, the SP still has a higher affinity for Hg due to the strong soft acid–soft base interactions by the form of Hg···N and Hg···S bonds [47].

### 2.4. Regeneration and Reusability

To minimize economic costs and reduce secondary pollution, the regeneration and reusability of the SP is also crucial for practical applications. Here, 0.05 M EDTA-2Na was used to elute the adsorbed Hg on the SP in the regeneration experiment. It can be seen that there is only a slight decrease in the removal (from 96.34% to 91.99%) and the uptake capacity (from 38.20 mg g^−1^ to 36.48 mg g^−1^) for Hg after five cycles (Figure S3). Therefore, the resulting SP has easy regeneration and good reproducibility.

### 2.5. Mathematical Modeling of Breakthrough Curves

**Figure 6:**
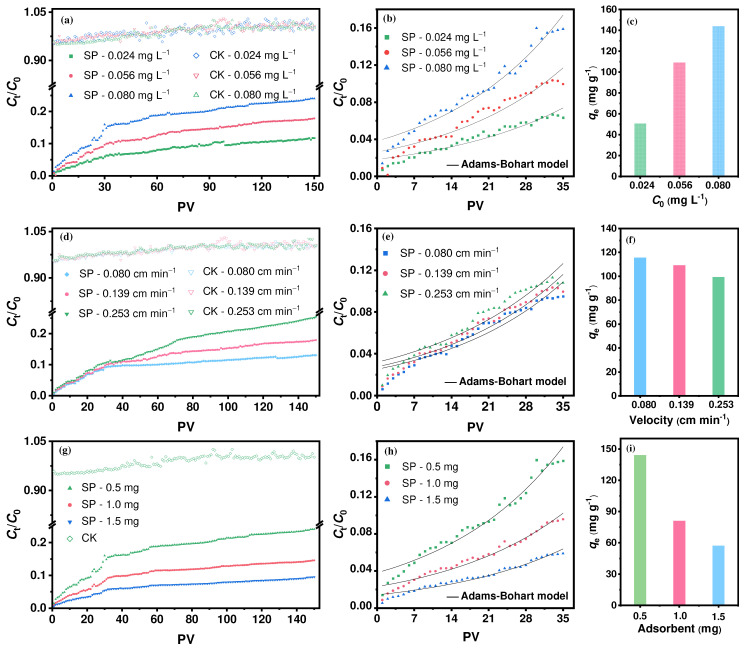
Effects of initial Hg concentration *C*_0_, influent pore velocity *ν*_p_, and adsorbent dosage *m* in groundwater on breakthrough curve of Hg adsorption on SP in fixed-bed column (25 ± 1 °C). Experimental conditions for (**a**–**c**): *C*_0_ = 0.024–0.080 mg L^−1^, *ν*_p_ = 0.139 cm min^−1^, and dosage *m* = 0.5 mg. Experimental conditions for (**d**–**f**): *ν*_p_ = 0.080–0.253 cm min^−1^, *C*_0_ = 0.056 mg L^−1^, and *m* = 0.5 mg. Experimental conditions for (**g**–**i**): *m* = 0.5–1.5 mg, *C*_0_ = 0. 080 mg L^−1^, and *ν*_p_ = 0.139 cm min^−1^.

The breakthrough curves of the fixed column show that *C*_t_/*C*_0_ generally increases with increasing pore volumes (PVs) (Figure 6). This may be due to the subsequent reduction of available sites on the SP as more groundwater flows through the column. After 150 PVs (the total groundwater influent volume is 1194 mL), the *C*_t_/*C*_0_ for the SP reached 9.50−25.37%. Control tests (CK) showed that quartz sand and column materials had minimal impact on Hg interception (<5%). The fitted results of four mathematical models are shown in Table 2 and Text S3. For 150 PVs, the Yan model, with higher *R*^2^ values (0.960–0.989) and lower error (SS and ARE), can describe the process more accurately under various operating conditions. Based on the fitting of the Yan model, the maximum adsorption capacity *q*_Y_ can be up to 3187 mg g^−1^, the *t*_b_ (95% breakthrough time) can reach 69.4 years, and the *p*_τ_ (pore volume of 50% breakthrough) ranges from 3646 to 11,531 PVs, indicating that the SP has an ultra-high uptake for mercury in groundwater. In addition, the Adams–Bohart model (*R*^2^ = 0.916−0.964) can better characterize the dynamic process of mercury interception during the initial phase (≤35 PVs).

In general, the operating variables in the column tests, including the initial concentration *C*_0_, influent pore velocity *ν*_p_, and adsorbent dosage *m*, significantly affected the Hg interception by the SP. As shown in Figure 6a–c and Table 2, a shorter breakthrough time was reached with a higher *C*_0_. When the *C*_0_ increased from 0.024 to 0.080 mg L^−1^, the *t*_b_ decreased from 42.7 years to 11.8 years, and the *q*_e_ increased from 50.0 to 144.0 mg g^−1^ (for 150 PVs). Mercury can be more easily transported from the aqueous solution to the SP surface due to the higher mass-transfer driving force caused by the high Hg concentration [64,72]. Similarly, the *t*_b_ decreased from 69.4 years to 7.16 years with an increasing *ν*_p_ from 0.080 to 0.253 cm min^−1^ (*q*_e_ dropped from 115.6 to 99.1mg g^−1^) (Figure 6d–f and Table 2). A high *ν*_p_ facilitates a reduction in the thickness of the mass-transfer film, which shortens the residence time for Hg from the external to the internal sites on the SP, resulting in low occupiable active sites on the SP [64,73]. With respect to the dosage *m*, a high *m* increased the *t*_b_ but inhibited the *q*_e_ (Figure 6g–i and Table 2). A high dosage provided more adsorption sites, leading to a relative surplus of available active sites on the SP [14,74].

### 2.6. Plausible Mechanism for Hg(II) Adsorption

**Figure 7:**
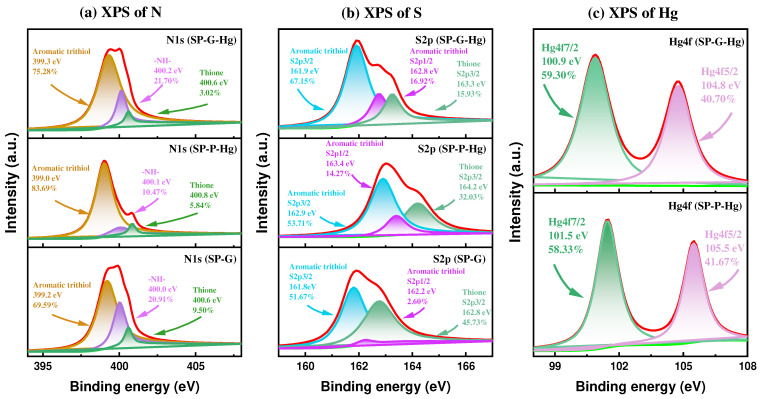
High-resolution XPS spectra of N1s (**a**), S2p (**b**), and Hg4f (**c**) for SP-G, SP-P-Hg (SP after adsorption in DI water with mercury), and SP-G-Hg.

To obtain insights into the mechanism of adsorption, the characterization methods SEM, XPS, and FT-IR were used. The highly consistent distribution and surface percentages of N, S atoms confirm the formation of the SP molecule (Figure 1, Figure 2c and Figure 5a). Compared to the SP, the presence of Hg (2.45%) and the consistent distribution of Hg, N, and S atoms demonstrate the complexation between Hg species (e.g., (Hg(OH)_2_, HgClOH, HgCl_2_, and ${\mathrm{HgOHCO}}_{3}^{-}$) in groundwater and the N, S composition in the SP, attributable to the strong soft acid–soft base interactions by the form of Hg···N and Hg···S bonds (Figure 5c). Signals centered at 288.13 eV, 532.29 eV, 162.03 eV, 399.63 eV, 199.91 eV, 347.87 eV, 1071.97 eV, and 101.08 eV indicate the presence of C1s, O1s, S2p, N1s, Cl2p, Ca2p, Nals, and Hg4f (Figure 2c). The doublet 4f peaks for Hg(II) suggest that mercury was successfully uploaded onto the SP surfaces (Figure 7c). For N1s high-resolution XPS spectra, compared to the SP, the lower content (from 9.50% to 3.02%) for the thione, the higher shift (from 400.0 eV to 400.2 eV) for –NH–, and the higher content (from 69.59% to 75.28%) for the aromatic trithiol also suggest the formation of Hg···N coordination bonds in the SP-G-Hg (Figure 7a). For S2p high-resolution XPS spectra, compared to the SP, the lower content for the thione (from 9.50% to 3.02%), the higher shift for –NH– (from 400.0 eV to 400.2 eV), and the higher content for the aromatic trithiol (from 69.59% to 75.28%) also suggest the formation of Hg···N coordination bonds in the SP-G-Hg (Figure 7b). For S2p high-resolution XPS spectra, compared to the SP, the lower content (from 45.73% to 15.93%) and higher shift (from 162.8 eV to 163.3 eV) for the thione S2p3/2, the higher content (from 2.60% to 16.92%) and the higher shift (from 162.2 eV to 162.8 eV) for aromatic trithiol S2p1/2, and the higher content (from 51.67% to 67.15%) and the higher shift (from 161.8 eV to 161.9 eV) for the aromatic trithiol S2p3/2 also suggest the formation of Hg···S coordination bonds in the SP-G-Hg (Figure 7b).

In the FTIR spectra (Figure 2a), the two undermining peaks around 1633 cm^−1^ and 1140 cm^−1^ indicate the formation of coordination bonds (metal–nitrogen and metal–sulfur) in the SP-G-Hg. The red shifts of aromatic trithiol (from 1482, 1240 and 780 cm^−1^ to 1420, 1235 and 774 cm^−1^) suggest that the coordinate bonds between mercury and ligands could stabilize the vibration of C_3_N_3_S_3_ rings in the SP-G-Hg. In addition, the SP can adsorb a small number of cations (Na^+^ and Ca^2+^) in groundwater without mercury duo to the electrostatic attraction driven by the electronegativity of the SP (Figure 5b). But under conditions of Hg-contaminated groundwater, mercury can replace these ions via ion exchange (Figure 5c), leading to a decrease in Na from 0.43% to 0.14%. For the formation of precipitates/co-precipitates, the Ca content shows an increasing trend. The adsorption mechanism can be described using a schematic diagram (Figure S4).

## 3. Materials and Methods

### 3.1. Materials and Characterization

All reagents used were of analytical grade or better without purification. Detailed information on the chemicals and characterizations is presented in the Supplementary Materials (Text S4).

### 3.2. Synthesis of SP

The supramolecular polymer (SP) was prepared according to our previous work, with some modifications [47]. Firstly, 1.773 g of trithiocyanuric acid was dispersed in 40 mL of water and heated at 70 °C via sonication for 1 h to form a homogeneous solution. Secondly, 1.261 g of melamine was mixed thoroughly with 30 mL of water accompanied by sonication for 0.5 h at 70 °C. Thirdly, the above two solutions were mixed together, transferred into a Teflon-lined autoclave, and kept at 100 °C for a further 2 h. The resulting light-yellow solid was then collected via filtration, purified with deionized water, and freeze dried (–60 °C, 48 h), which is referred to as the SP. Figure S5 shows photographs of the raw materials and the as-prepared SP, together with the proposed synthesis route of the SP.

### 3.3. Batch Experiments

For batch sorption procedures of mercury in groundwater, 10 mg of the SP was dispersed into 30 mL of the desired Hg^2+^ simulated groundwater (containing 234.3 mg L^−1^ C1^−^, 96.0 mg L^−1^ ${\mathrm{SO}}_{4}^{2-}$, 183.0 mg L^−1^ ${\mathrm{HCO}}_{3}^{-}$, 230.0 mg L^−1^ Na^+^, and 32.0 mg L^−1^ Ca^2+^) in sealed 50 mL PTFE vials. The initial pH of the simulated groundwater was 8.08 ± 0.2. HCl and NaOH were used to adjust the pH. The suspension was shaken under 200 rpm at 25 °C and then filtered using 0.22 μm PTFE. The concentration of Hg in the filtrate was determined using atomic fluorescence spectrometry (AFS), and that of the other metal ions was detected using inductively coupled plasma optical emission spectrometry (ICP-OES). For sorption kinetics, the initial Hg^2+^ concentration was 0.08 mg L^−1^, the sorbent dosage was 2.0 mg L^−1^, and the experiment was conducted under pH 6.0. In sorption isotherms, the Hg^2+^ concentrations were 0.01–1.50 mg L^−1^, and the other experimental conditions were the same as above. The effects of environmental factors, including the pH, DOM, ionic strength, and coexisting ions (Cd, Zn, Pb, Cr, Cu, and Ni), were also determined. All experiments were carried out in triplicate. Control tests without the SP (called CK) showed that the Hg loss during the process was <5%. Calculations of the removal efficiency *R* (%) and the adsorption capacity *q_e_* (mg g^−1^) are shown in Text S5. 

### 3.4. Column Tests

Continuous fixed-bed column tests were performed to investigate the remediation performance of the SP in permeable reactive barriers (PRBs) for mercury removal in simulated groundwater. The column experiments were conducted in a glass column (006EZ-10-25-AF, Omnifit, Amersham, UK). Under the driving force of the pump, the simulated groundwater was continuously injected into the column and flowed through layers of quartz sands, SP sorbents, quartz sands, and glass wool in sequence from top to bottom (Figure S6). The effluent was periodically collected by the automated sampler for subsequent analysis. The analysis method for metal ion concentration was the same as the batch experiment. The control column (quartz sands/glass wool) test was conducted under identical conditions. Several operating conditions of the column test, such as the influent Hg^2+^ concentration *C*_0_, pore velocity *ν*_p_, and sorbent dosage *m*, were studied to investigate the effects. The breakthrough time (*t*_b_) and the total Hg sorbed in the column (*q*_t_, mg g^−1^) were adopted and are presented in Text S6 [14].

### 3.5. Mathematical Models for Breakthrough Curves

Four mathematical models were used to simulate the breakthrough curves in order to describe the dynamic Hg concentration change of the effluent, including the Adams–Bohart [75], Thomas [76], Yan [77], and Yoon–Nelson models [78]. The models can be expressed as Equations (1)–(4), respectively.

(1) $$\frac{C_{t}}{C_{0}}=\exp\left(\frac{K_{\mathrm{AB}}C_{0}V_{0}}{Q}n-\frac{K_{\mathrm{AB}}N_{0}H}{\nu_{p}}\right)$$

(2) $$\frac{C_{t}}{C_{0}}=\frac{1}{1+\exp\left(\frac{1000K_{\mathrm{Th}}q_{\mathrm{Th}}m-V_{0}K_{\mathrm{Th}}C_{0}n}{1000Q}\right)}$$

(3) $$\frac{C_{t}}{C_{0}}=1-\frac{1}{1+\left(\frac{C_{0}V_{0}n}{1000q_{Y}m}\right)}$$

(4) $$\frac{C_{t}}{C_{0}}=\frac{1}{1+\exp\left(\frac{K_{\mathrm{YN}}V_{0}p_{\tau }-K_{\mathrm{YN}}V_{0}n}{Q}\right)}$$

Detailed information on *C*_t_/*C*_0_, *K*_AB_, *C*_0_, *V*_0_, *H*, *Q*, *n*, *ν*_p_, *K*_Th_, *q*_Th_, *m*, *q*_Y_, *p*_τ_, and *K*_YN_ in the above models is given in Text S3.

## 4. Conclusions

Our study demonstrated that the as-synthesized SP with excellent applicability can effectively remove low-concentration mercury in high-salinity groundwater despite unfavorable conditions such as organic molecules, coexisting metal ions, and high ionic strength. The adsorption performance fitted well with the pseudo-second-order kinetic model (*R*^2^ = 0.9996) and the Langmuir isotherm model (*R*^2^ = 0.9803), signifying that the process belongs to chemisorption and monolayer sorption. The maximum adsorption capacity *q*_m_ can reach 926.1 ± 165.3 mg g^−1^ due to the rich –SH and –NH_2_ in the SP. Furthermore, the SP can serve as an excellent PRB for intercepting mercury in groundwater, and the Yan model and Adams–Bohart model can better describe the breakthrough curves. Further mechanistic studies suggest that complexation, electrostatic attraction, ion exchange, and precipitation play a major role. These practical performances, including excellent selectivity, high uptake capacity, superior anti-interference ability, and longer breakthrough time, support that the SP will be a good candidate for the practical PRB remediation of mercury-contaminated groundwater.

## Figures and Tables

**Table 1:** Comparison of mercury uptake using different adsorbents.

Adsorbents	*q*_max_ (mg g^−1^)	Ref.
rGO-p(C_3_N_3_S_3_)/Fe_3_O_4_	400.0	[28]
MoS_2_/Fe_3_O_4_	425.5	[63]
SGO/Fe–Mn	112.03	[64]
PSAC-S	136	[23]
CCTNT	199.80	[24]
BCS	4.30	[2]
MPTAPs)	211	[36]
Zr-MSA/DCS	312.4	[25]
SP	926 ± 165	Present work

**Table 2:** Fitting parameters of Adams–Bohart and Yan models for Hg adsorption by SP in column test.

Variables	Yan Model							Adams–Bohart Model				
	*K*_Y_	*q*_Y_	*R* ^2^	*SS*	*ARE*	*t*_b_	*p*_τ_	*K*_AB_(×10^−3^)	*N*_0_	*R* ^2^	*SS*	*ARE*
		(mg g^−1^)		(×10^−5^)	(%)	(Year)		(L min^−1^ mg^−1^)	(mg L^−1^)		(×10^−5^)	(%)
*C*_0_ (mg L^−1^) ^a^												
0.080	0.485	3187	0.962	11.8	8.29	11.8	3646	7.50	2.75	0.957	5.38	13.8
0.056	0.498	2344	0.983	3.17	14.8	24.5	6142	10.6	2.18	0.916	6.03	62.6
0.024	0.520	1870	0.982	1.42	6.08	42.7	8350	22.8	1.11	0.926	1.87	16.5
*ν*_p_ (cm min^−1^) ^b^												
0.253	0.383	1764	0.989	4.03	7.94	7.16	3927	17.9	2.22	0.922	5.67	17.4
0.139	0.498	2344	0.983	3.17	14.8	24.5	6142	10.6	2.18	0.916	6.03	62.6
0.080	0.603	2984	0.977	7.62	29.0	69.4	11,531	6.01	2.22	0.920	4.76	23.3
*m* (mg) ^c^												
0.5	0.485	3187	0.962	11.8	8.29	11.8	3646	7.50	2.75	0.957	5.38	13.8
1.0	0.504	2474	0.960	8.46	56.4	26.35	8062	7.33	2.82	0.963	1.44	13.32
1.5	0.576	1759	0.967	1.03	16.9	47.92	9705	7.37	2.80	0.964	5.56	13.29

Note: The results of the Yan model and Adams–Bohart model were fitted for 150 PVs and 35 PVs, respectively. *C*_0_ (mg L^−1^) is the influent concentration of Hg^2+^; *v*_p_ (cm min^−1^) is the pore velocity of groundwater, i.e., the distance flowed per minute; *m* (mg) is the amount of material used; *K*_Y_ and *q*_Y_ (mg g^−1^) are the constant and the maximum adsorption capacity for the Yan model, respectively; *t*_b_ (h) is the time of 95% breakthrough (*C*_t_/*C*_0_ = 0.95); *p*_τ_ is the number of pore volumes at 50% adsorbate breakthrough (*C*_t_/*C*_0_ = 0.5) for the Yan model; *K*_AB_ (L min^−1^ mg^−1^) and *N*_0_ (mg L^−1^) are the rate constant and saturated adsorption capacity per column volume for the Adams–Bohart model, respectively; *R*^2^, *SS*, and *ARE* are the coefficients of determination, least sum of squares, and average relative error, respectively. Detailed information is shown in Text S3. Experimental conditions: ^a^: *ν_p_* = 0.139 cm min^−1^, *m* = 0.5 mg, and 25 ± 1 °C; ^b^: *C*_0_ = 0.056 mg L^−1^, *m* = 0.5 mg, and 25 ± 1 °C; ^c^: *C*_0_ = 0.080 mg L^−1^, *ν*_p_ = 0.139 cm min^−1^, and 25 ± 1 °C.

## Data Availability

The original contributions presented in this study are included in the article/Supplementary Materials. Further inquiries can be directed to the corresponding author.

## Supplementary Materials

The following supporting information can be downloaded at https://www.mdpi.com/article/10.3390/molecules30071406/s1: Text S1. The pseudo-first-order and pseudo-second-order models. Text S2. The Langmuir isotherm and Freundlich isotherm models. Text S3. The four mathematical models for breakthrough curves. Text S4. Materials and instrumentation. Text S5. The adsorption capacity and removal efficiency. Text S6. The time of 95% breakthrough. Figure S1. SEM images of SP. Figure S2. Mercury sorption kinetics in simulated groundwater (*m* = 2.0 mg L^−1^ adsorbent, *C*_0_ = 0.08 mg L^−1^; reaction time was 5 h) and adsorption removal efficiencies (R) at multiple initial Hg^2+^ concentrations after mercury uptake from simulated groundwater (*m* = 2.0 mg L^−1^, *C*_0_ = 0.01–1.50 mg L^−1^; reaction time was 5 h). Fitting of the isotherms (T = 308 K) and (d) (T = 318 K) in the simulated groundwater for mercury adsorption onto the SP (*m* = 2.0 mg L^−1^; *C*_0_ = 0.01–1.50 mg L^−1^). Figure S3. Recycling performance of mercury onto SP (*m* = 2.0 mg L^−1^; T = 298 K; *C*_0_ = 0.08 mg L^−1^). Figure S4. Underlying adsorption mechanisms of SP for Hg species in simulated groundwater. Figure S5. The proposed route of synthesis and the chemical formula of the SP; the photographs of raw materials and the as-prepared SP. Figure S6. Scheme of the fixed-bed column test system. Table S1. Pseudo-first-order and pseudo-second-order kinetic models used for simulating Hg sorption kinetic data and the resulting fitting parameters. Table S2. Fitting parameters of Langmuir and Freundlich isotherm models for Hg sorption. Table S3. Thermodynamic parameters for mercury adsorption onto SP at different temperatures (*m* = 2.0 mg L^−1^; *C*_0_ = 0.08 mg L^−1^). Table S4. Fitting parameters of Thomas and Yoon–Nelson models for Hg adsorption by SP in column test.
