# Supplementary material for: Remediation of Hg-Contaminated Groundwater via Adsorption on Supramolecular Polymers in Batch Process and Column Test
Source: Molecules. 2025 Mar 21;30(7):1406. doi: 10.3390/molecules30071406 (PMC11990362; doi:10.3390/molecules30071406)
Supplement: Supplementary file 1 [file molecules-30-01406-s001.zip › molecules-3416890-supplementary.pdf]

Remediation of Hg-Contaminated Groundwater via Adsorption on Supramolecular Polymers in Batch Process and Column Test

Zongwu Wang [1], Wei Liu [1], Xiaoyan Sun [1], Qing Zhang [2], Jiapu Ji [1], Yimeng Yan [1] and Jianhui Sun [3,*]

[1] Department of Environment Engineering, Yellow River Conservancy Technical Institute, Kaifeng Engineering Research Center for Municipal Wastewater Treatment, Kaifeng 475004, China
[2] Product Quality Inspection and Testing Center of Kaifeng, Kaifeng 475004, China
[3] MOE Key Laboratory of Yellow River and Huai River Water Environmental and Pollution Control, School of Environment, Henan Normal University, Xinxiang 453007, China
***** Correspondence: sunjh@htu.edu.cn

Total number of pages of Supporting Information: **11**

Number of Texts in Supporting Information: **6**

Number of Tables in Supporting Information: **4**

Number of Figures in Supporting Information: **6**

Text S1

The pseudo-first-order and the pseudo-second-order models are described as Eqs. (S1) and (S2), respectively:

$$\ln\left(q_{\mathrm{e}}-q_{\mathrm{t}}\right)=\ln q_{\mathrm{e}}-K_1 t \tag{S1}$$

$$\frac{t}{q_{\mathrm{t}}}=\frac{t}{q_{\mathrm{e}}}+\frac{1}{K_2 q_{\mathrm{e}}^2} \tag{S2}$$

where q_{t} (mg g^{-1}) is uptake of Hg^{2+} by adsorbents at time t (min), $q = (C_0 - C_{\mathrm{e}}) \times V/m$, C_0 and C_{e} are the initial and equilibrium concentrations of Hg^{2+}. V is the volume of the solution (L), and m is the mass of adsorbent (mg), q_{e} is the uptake of Hg^{2+} at equilibrium, K_1 is the rate constant of the pseudo-first-order sorption, K_2 is the pseudo-second-order sorption rate constant. The governing equations of the two models are integrated by applying the boundary conditions $q = 0$ (t = 0). Pseudo-first-order was based on the theory of membrane diffusion, and the adsorption rate is related to the difference between the equilibrium adsorption uptake and the adsorption uptake. Pseudo-second-order was established on the adsorption rate limiting step, involving electron sharing or electron transfer between the adsorbate and the adsorbent, representing chemical adsorption. All kinetic parameters were calculated through nonlinear regression using OriginLab 2021 software program.

Text S2

The Langmuir isotherm model and Freundlich isotherm model (Eq. S3 and S4) are respectively as the following:

$$q_{\mathrm{e}}=\frac{q_{\mathrm{m}} b_{\mathrm{L}} C_{\mathrm{e}}}{1+b_{\mathrm{L}} C_{\mathrm{e}}} \tag{S3}$$

$$q_{\mathrm{e}}=K_{\mathrm{f}} C_{\mathrm{e}}^{n} \tag{S4}$$

Where q_{e} is the uptake of Hg Hg^{2+} at equilibrium (mg g^{-1}), C_{e} is the equilibrium aqueous mercury concentration (mg L^{-1}). Langmuir isotherm: q_{m} is the maximum sorption capacity, b_{L} is the Langmuir constant related to the free energy of adsorption. Freundlich isotherm: K_{f} is a constant related to the adsorption capacity of the sorbent, n is the adsorption intensity or the heterogeneity of the sorbent. Langmuir isotherm model depicts monolayer sorption, and Freundlich isotherm model describes nonideal sorption on heterogeneous surfaces. All isotherm parameters were calculated through nonlinear regression using OriginLab 2021 software program.

Text S3

(1) Adams-Bohart model

$$\frac{C_\mathrm{t}}{C_0} = \exp\left(\frac{K_\mathrm{AB}C_0V_0}{Q}n - \frac{K_\mathrm{AB}N_0H}{v_\mathrm{p}}\right) \ (C_\mathrm{t}/C_0<0.5) \tag{S5}$$

Where K_{AB} (L min^{-1} mg^{-1}) is the Adams-Bohart model rate constant; N_0 (mg L^{-1}) is the saturated adsorption capacity per column volume; H (cm) is the bed depth of the column; v_p (cm min^{-1}) is the pore velocity; n is the number of pore volumes; V_0 (mL) is one pore volume of the packed column (7.96 mL in this study); and Q (mL min^{-1}) is the volumetric flow rate; C_0 (mg L^{-1}) and C_t (mg L^{-1}) are the mercury concentrations in the influent and at time t (min), respectively. $t = n \times V_0/Q$. The values of K_{AB} and N_0 can be evaluated from a plot of C_t/C_0 against n by non-linear regression analysis.

(2) Thomas model

$$\frac{C_\mathrm{t}}{C_0} = \frac{1}{1+\exp\left(\frac{1000K_\mathrm{Th}q_\mathrm{Th}m - V_0K_\mathrm{Th}C_0n}{1000Q}\right)} \tag{S6}$$

Where K_{Th} (mL min^{-1} mg^{-1}) is the Thomas rate constant; q_{Th} (mg g^{-1}) is the equilibrium sorption capacity; m (g) is the amount of sorbent in the column; other parameters have the same meaning as above. The values of K_{Th} and q_{Th} can be determined from a plot of C_t/C_0 *vs*. n at given experimental conditions using non-linear regression analysis.

(3) Yan model

$$\frac{C_\mathrm{t}}{C_0} = 1 - \frac{1}{1+\left(\frac{C_0V_0n}{1000q_\mathrm{Y}m}\right)} \tag{S7}$$

Where q_Y (mg g^{-1}) is the maximum adsorption capacity; K_Y is the constant of Yan model; other parameters have the same meaning as above. The values of K_Y and q_Y can be evaluated from a plot of C_t/C_0 against n using non-linear regression analysis.

(3) Yoon-Nelson model

$$\frac{C_\mathrm{t}}{C_0} = \frac{1}{1+\exp\left(\frac{K_\mathrm{YN}V_0p_\tau - K_\mathrm{YN}V_0n}{Q}\right)} \tag{S8}$$

Where K_{YN} (min^{-1}) is the Yoon-Nelson kinetic constant; and p_{τ} represents the number of pore volumes at time τ (the contact time required 50% adsorbate breakthrough, C_t/C_0 = 0.5); other parameters have the same meaning as above. The values of K_{YN} and p_{τ} can be evaluated from a plot of C_t/C_0 *vs.* n using non-linear regression analysis as the values of C_t/C_0 are within 0.05–0.95.

Text S4

Materials: Trithiocyanuric acid ($C_3H_3N_3S_3$) and Melamine ($C_3H_6N_6$) were purchased from Aladdin Reagents. Mercury nitrate monohydrate ($Hg(NO_3)_2 \cdot H_2O$) was obtained from Sinopharm Chemical Reagent Co. Ltd. (Shanghai, China). Reference material (RM) of Hg (1000 mg L^{-1} in 5% HNO_3) was obtained from Tan-Mo Technology Co., Ltd (Beijing, China). NaCl, Na_2SO_4, $CaCl_2$, $NaHCO_3$ and Quartz sand (20~40 mush) were procured from Damao chemical reagent factory (Tianjin, China). Potassium hydroxide (KOH) (AR), potassium borohy-dride (KBH_4) (AR), glucose (98%), and humic acid (98%) were obtained from Macklin Reagent Co. Ltd. (Shanghai, China). Hydrochloric acid (HCl) and sodium hydroxide (NaOH) (AR) were obtained from Tianjin Chemical Reagent Technology (Tianjin, China). All solutions were prepared with deionized water (18 MΩ·cm).

Instrumentation: Structure and morphology were observed by scanning electron microscopy (SEM) (Sigma300, Carl Zeiss) and transmission electron microscopy (TEM) (JEM-2100, JEOL). The surface atomic ratio and the binding energy of C1s, N1s, S2p, Na1s, Ca2p, O1s, C1s and Hg4f were analyzed by X-ray photoelectron spectrometer (XPS) (K-alhpa, Thermo Fisher) with Al-Kα radiation (pass energy 150.0 eV). X-ray diffraction (XRD) pattern was collected on Bruker D8-AXS. Organic functional groups were determined by fourier transform infrared spectrometer (FT-IR) (iS10, Thermo Fisher). The Zeta potential and particle size distribution were determined using a Zeta potential and nanoparticle size analyzer (Nanotrac wave II, Microtrac). N_2 adsorption-desorption isotherms were performed using Micromeritics ASAP2020. The pH was measured with a pH meter (FE28 stander, Mettler Toledo). Mercury and calcium ion speciation in simulated groundwater was conducted using Visual MINTEQ 3.1.

Text S5

The adsorption capacity q_e (mg g^{-1}) and removal efficiency of adsorbent was calculated according the following equations:

$$q_{\mathrm{e}} = \frac{(C_0 - C_e)V}{m} \tag{S9}$$

$$R\% = \frac{C_0 - C_e}{C_0} \times 100\% \tag{S10}$$

Where V (L) is the volume of the water solution, C_0 and C_e (mg L^{-1}) are the initial and equilibrium concentrations of the metal ions, m (g) is the mass of adsorbent used. All experiments were performed in triplicate.

Text S6

The t_b is the time of 95% breakthrough (C_t/C_0 = 0.95). The total amount of mercury sorbed in the column (q_t, mg g^{-1}) can be calculated according to the following equation (S7) at a given influent mercury concentration (C_0):

$$q_t = \int_0^{V_t} \frac{C_0 - C_t}{m} dV \tag{S11}$$

Where C_t (mg g^{-1}) is the effluent Hg^{2+} concentration, V (L) is the volume of mercury contaminated groundwater, V_t (L) is the total volume of groundwater when the effluent mercury concentration reaches C_t, and m (g) is the mass of sorbent in column.

Table S1

Pseudo-first-order and pseudo-second-order kinetic models used for simulating Hg sorption kinetic data and the resulting fitting parameters.

Kinetic model	Parameters	Values
Pseudo-first-order $\ln(q_e - q_t) = \ln q_e - K_1 t$	K_1 (min^{-1})	(1.82±0.21)×10^{-2}
	q_e (mg g^{-1})	33.04±2.23
	R^2	0.9262
Pseudo-second-order $\frac{t}{q_t} = \frac{t}{q_e} + \frac{1}{K_2 q_e^2}$	K_2 (g (mg·min)$^{-1}$)	(2.54±0.16)×10^{-3}
	q_e (mg g^{-1})	39.22±0.31
	R^2	0.9996

Note: q_t (mg g^{-1}) is the uptake of Hg^{2+} by the sorbents at time t (min), q_e is the equilibrium uptake of Hg^{2+}, $q_e = (C_0 - C_e) \times V/m$, where C_0 and C_e are the initial and equilibrium concentrations of Hg^{2+} (mg L^{-1}). V is the volume of the solution (L), and m is the mass of the adsorbent (mg), K_1 is the pseudo-first-order sorption rate constant, K_2 is the pseudo-second-order sorption rate constant, h_1 is the initial rate of pseudo-first-order sorption, h_2 is the initial rate of pseudo-second-order sorption. The governing equations of the two models are integrated by applying the boundary conditions $q = 0$ at $t = 0$. R^2 is the coefficient of determination. All kinetic parameters were calculated through nonlinear regression using Origin 2021 software program.

Table S2

Fitting parameters of Langmuir and Freundlich isotherm models for Hg sorption.

Adsorption isotherm	Parameters	SP
Langmuir model $q_e = \frac{q_m b_L C_e}{1 + b_L C_e}$	q_m (mg g^{-1})	926.1 ± 165.3
	b_L (L mg^{-1})	12.60 ± 3.04
	R^2	0.9803
Freundlich model $q_e = K_f C_e^n$	K_f (mg g^{-1})/(mg $L^{-1})^n$	(3.30 ± 0.449) ×10^2
	n	0.76 ± 0.03
	R^2	0.9780

Note: q_e is the equilibrium Hg^{2+} uptake (mg g^{-1}), C_e is the equilibrium aqueous Hg^{2+} concentration (mg L^{-1}). Langmuir isotherm: q_m is the maximum sorption capacity, b_L is the Langmuir constant related to the free energy of adsorption. Freundlich isotherm: K_f is a constant related to the adsorption capacity of the sorbent, n is the adsorption intensity or the heterogeneity of the sorbent. R^2 is the coefficient of determination calculated. All isotherm parameters were calculated through by non-linear regression using Origin 2021 software program.

Table S3

Thermodynamic parameters for mercury adsorption onto SP at different temperatures (m = 2.0 mg L^{-1}, C_0 = 0.08 mg L^{-1}).

ΔG(kJ mol^{-1})			ΔH(kJ mol^{-1})	ΔS(J mol^{-1} K^{-1})
298 K	308 K	318 K		
-23.50	-24.39	-25.27	2.92	88.65

Note: The relevant thermodynamic parameters including the Gibbs function change ΔG, the enthalpy changes ΔH and the entropy changes ΔS calculated by the equations of $\ln \frac{q_e}{C_e} = \frac{\Delta S}{R} - \frac{\Delta H}{R} \times \frac{1}{T}$ and $\Delta G = \Delta H - T \times \Delta S$.

Table S4

Fitting parameters of Thomas and Yoon-Nelson models for Hg adsorption by SP in column test.

Variables	Thomas model					Yoon-Nelson model				
	K_{Th} (mL min^{-1} mg^{-1})	q_{Th} (mg g^{-1})	R^2	SS ($\times10^{-4}$)	ARE (%)	K_{YN}($\times10^{-4}$) (min^{-1})	p_τ	R^2	SS ($\times10^{-6}$)	ARE (%)
C_0 (mg L^{-1})[a]										
0.080	1.28	360.6	0.761	7.51	25.7	1.01	285.3	0.761	7.51	25.7
0.056	1.93	290.0	0.814	3.46	52.9	1.07	326.8	0.819	3.46	52.9
0.024	4.24	155.2	0.822	1.29	23.9	1.02	407.1	0.821	1.40	23.9
v_p (cm min^{-1})[b]										
0.253	4.49	216.2	0.886	4.78	24.1	2.50	244.1	0.886	4.78	24.1
0.139	1.93	290.0	0.814	3.46	52.9	1.07	326.8	0.819	3.46	52.9
0.080	0.814	412.2	0.701	2.35	27.6	0.50	457.5	0.701	2.35	27.6
m (mg)[c]										
0.5	1.28	360.6	0.761	7.51	25.7	1.01	285.3	0.761	7.51	25.7
1.0	2.12	249.0	0.754	2.80	25.8	0.93	394.0	0.754	2.80	25.8
1.5	2.10	200.0	0.784	0.98	24.9	0.92	484.8	0.784	0.98	24.9

Note: The results of Thomas and Yoon-Nelson models were fitted for 150 PVs. p_τ is the number of pore volumes at 50% adsorbate breakthrough (C_t/C_0 = 0.5); R^2, SS and ARE are coefficients of determination, least sum of squares, and average relative error, respectively. Detailed information is shown in Text S4.

Experimental conditions:

[a]: v_p = 0.139 cm min^{-1}, m = 0.5 mg, 25 ± 1 °C;

[b]: C_0 = 0.056 mg L^{-1}, m = 0.5 mg, 25 ± 1 °C;

[c]: C_0 = 0.080 mg L^{-1}, v_p = 0.139 cm min^{-1}, 25 ± 1 °C.

Figure S1

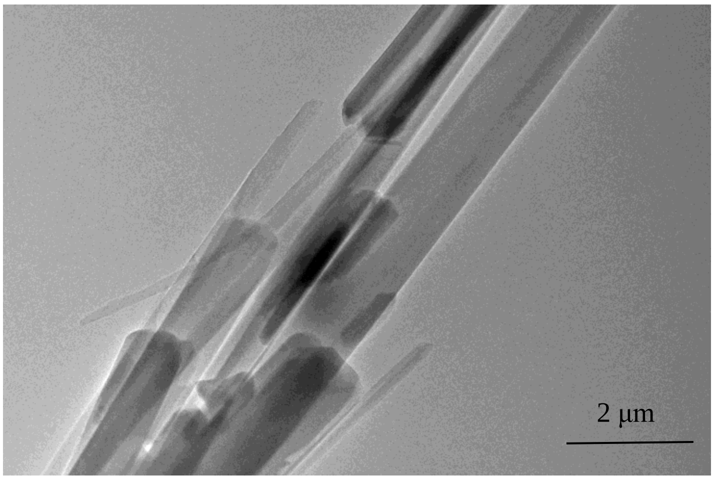

Figure S1. SEM images of SP.

Figure S2

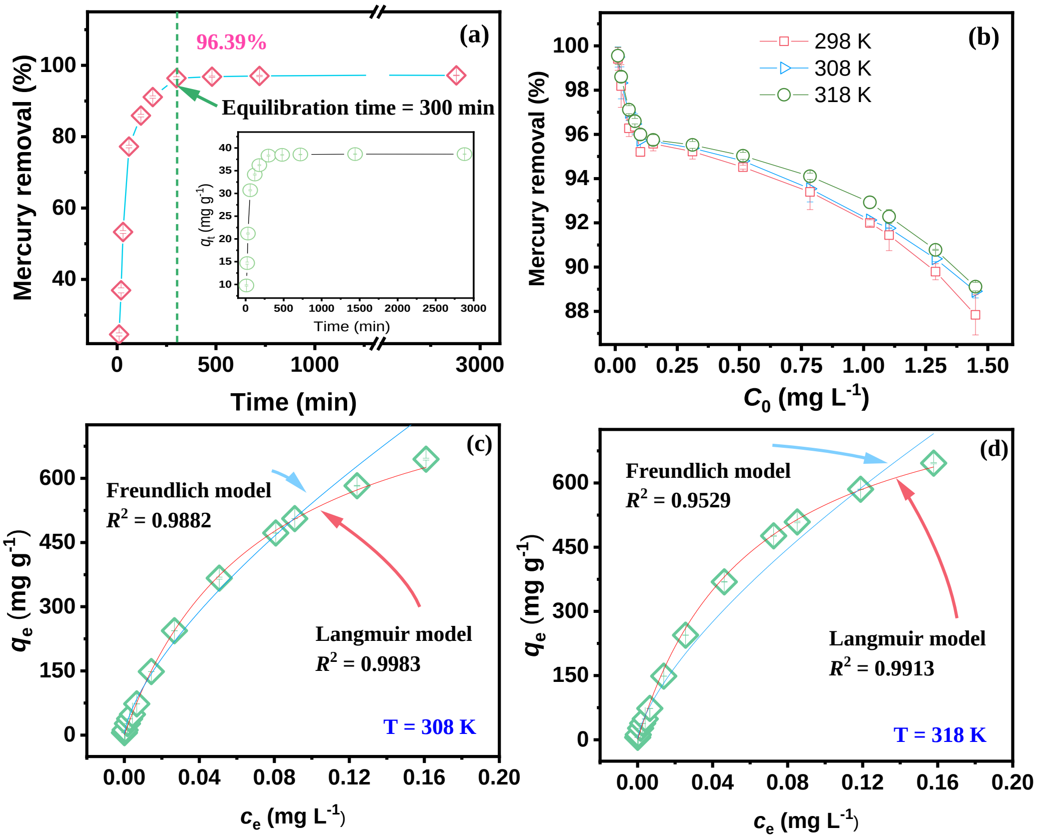

Figure S2. (a) Mercury sorption kinetics in simulated groundwater (m = 2.0 mg L^{-1} adsorbent, C_0 = 0.08 mg L^{-1}, reaction time was 5 h) and **(b)** adsorption removal efficiencies (R) at multiple initial Hg^{2+} concentrations after mercury uptake from simulated groundwater (m = 2.0 mg L^{-1}, C_0 = 0.01-1.50 mg L^{-1}, reaction time was 5 h). Fitting of the isotherms **(c)** (T = 308 K) and **(d)** (T = 318 K) in the simulated groundwater for mercury adsorption onto SP (m = 2.0 mg L^{-1}, C_0 = 0.01-1.50 mg L^{-1}).

Figure S3

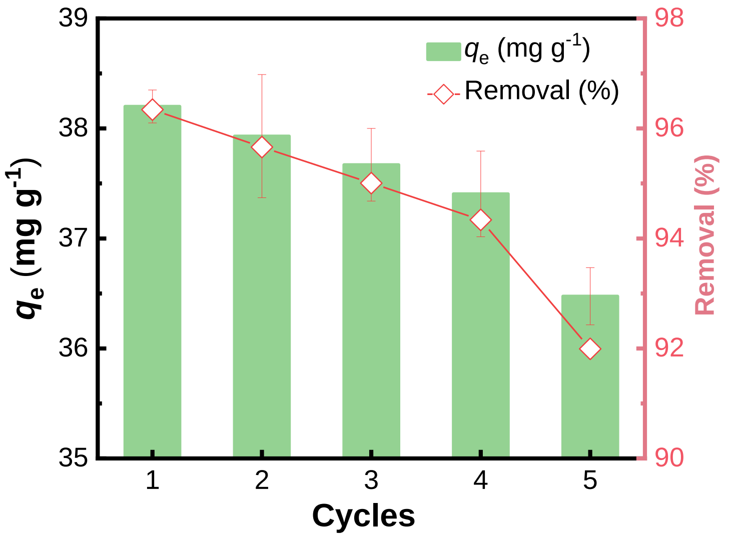

Figure S3. Recycles performance of mercury onto SP (m = 2.0 mg L^{-1}, T = 298 K, C_0 = 0.08 mg L^{-1})

Figure S4

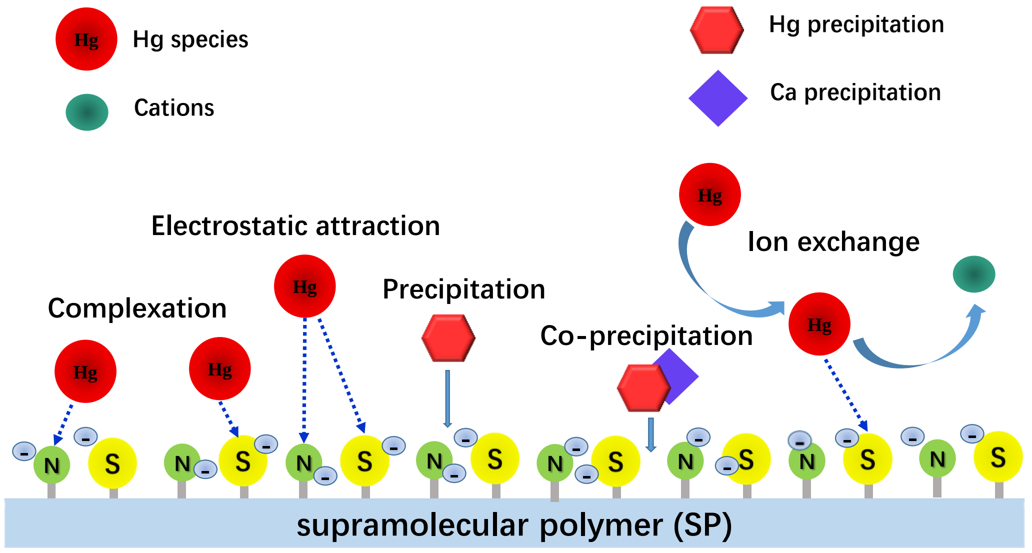

Figure S4. Underlying adsorption mechanisms of SP for Hg species in simulated groundwater.

Figure S5

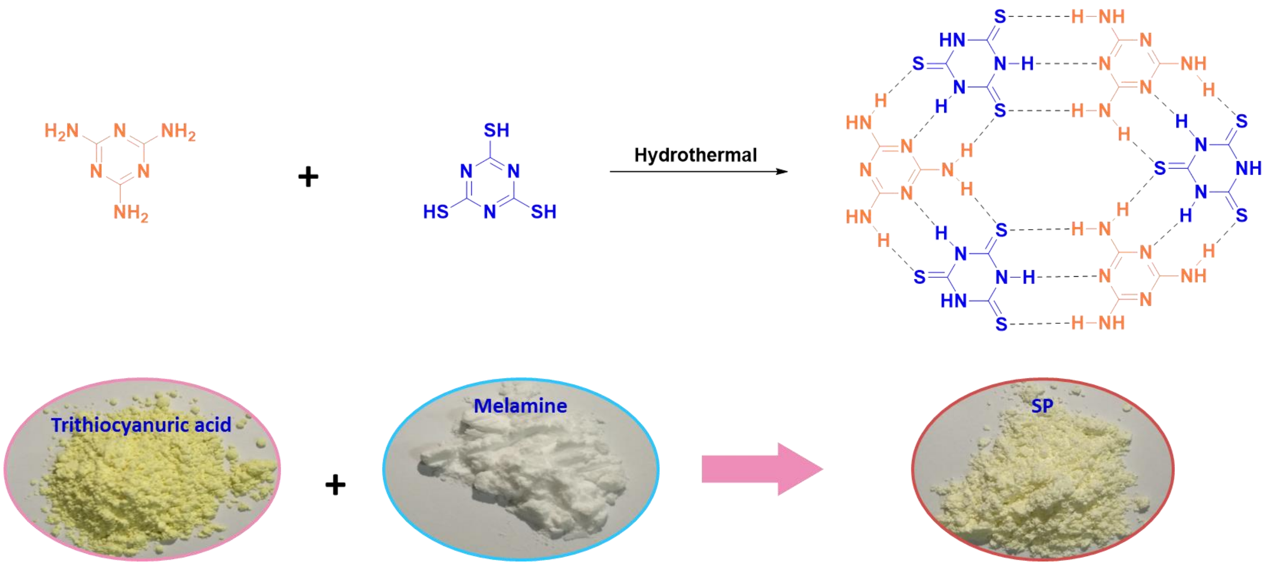

Figure S5. The proposed route of the synthesis and the chemical formula of SP; the photographs of raw materials and as-prepared SP.

Figure S6

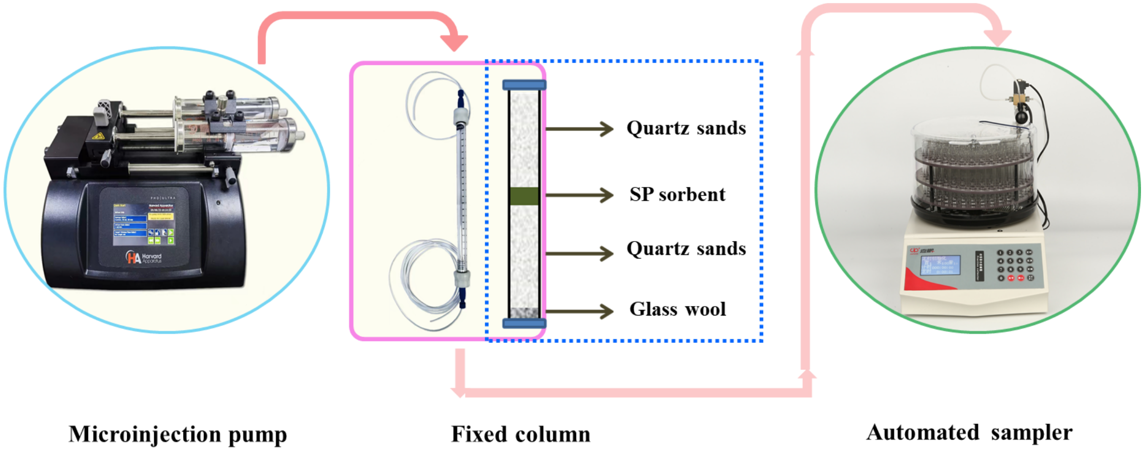

Figure S6. Scheme of the fixed-bed column test system.

Note: The four layers inside the column from bottom to top are: 0.2 g glass wool (height = 0.3 cm); quartz sands (porosity = 0.32, and height = 10.0 cm); 0.5 mg SP (mixed with quartz sands, height = 0.3 cm); quartz sands (porosity = 0.32, and height = 11.4 cm), the pore volume (PV) of the packed column was determined to be 7.96 mL. The microinjection pump (Harvard Apparatus PHD/ULTRA, USA) and the automated sampler (Jiapeng BSZ–160, Shanghai, China) were employed. The quartz sands was pretreated following the reported approach.
